# Peritoneal Adhesions Caused by Muscular Trauma

**Published:** 1903-09-19

**Authors:** 


### PERITONEAL ADHESIONS CAUSED BY MUSCULAR TRAUMA.

THE presence of peritoneal adhesions affecting mainly the large bowel and occurring especially in old people is a sufficiently familiar thing in post-mortem work, and the usual tendency is probably to regard the adhesions either as the result of normal processes or as the effect of chronic constipation. Dr. Byron Robinson,[1] however, puts forward the theory that they are caused by muscular trauma. His theory is based upon anatomical observations which he has made in the course of 485 autopsies. Thus he has noticed that the commonest places for these adhesions to occur are: (1) the ventral surfaces of the psoas muscles; (2) the ventral surfaces of the crura of the diaphragm; (3) the external lateral surfaces of the ascending and descending colon; (4) the lateral abdominal surfaces of the diaphragm close to its costal attachments; (5) the surface of the levator ani and coccygeus muscles. He believes that where a segment of the intestinal tract lies within the range of action of a strong muscle, like the psoas, the trauma of the muscle on the bowel segment is able to cause the intestinal bacteria or their products to pass through the bowel wall and set up a local peritonitis resulting in the formation of adhesions. He further states that the most marked adhesions are to be found in those situations where a portion of intestine is fixed within the widest range of action of a muscle, the most striking example being the sigmoid flexure which is liable to injury by the left psoas muscle; mesosigmoiditis, as shown by the presence of adhesions, being present in 80 per cent. of adult subjects. In 60 post-mortem examinations of children practically no peritoneal adhesions were found in those who were under three years of age.

[1] Medical Record, August 29, 1903.

